# On the Formation of Abscesses External to the Bone after Fractures by Contre-coup of the Long Bones and Compound Luxations

**Published:** 1846-11

**Authors:** 


*On the formation of Abscesses external to the Bone after fractures by contre-coup of the Long Bones and compound luxations.* By M. Laugier.—In the last number of the *Archives Généralés*, M. Laugier has published a memoir under the foregoing title :—When in a luxation or a fracture the articular extremity or a fragment of the bone perforates the skin, it might be supposed that the greatest injury to the parts would be inflicted, and the chances of suppurative inflammation be greatest on the side where the integuments are perforated; but on the contrary, when an abscess forms, it is constantly on the opposite side. This M. Laugier explains as follows:

On examining the extent to which the soft parts are separated from the surface of the bone on the side towards which the displacement occurs and on the opposite side, it will be seen that the separation is always greater in the latter situation, especially in displacements by *contre-coup.* Thus, to take a particular case, fractures of the leg by *contre-coup* usually occur at the union of the lower and the middle third of the tibia, and are generally oblique outwards, and from above downwards. Suppose the upper fragment of the tibia, in such a fracture, perforates the skin and protrudes internally to the extent of an inch, on examining, before the fracture is reduced, on what side of the bone the disturbance of the soft parts is most extensive, it will be found to be so on the outer side of the bone. Doubtless on the inner side the integuments are perforated and the bone projects, but the soft parts remain adherent to the bone above the portion which actually projects. On the external surface of the bone, on the contrary, the separation of the soft parts from it and the laceration of the cellular tissue will have occurred to the extent of from two-and-a-half to three inches when the bone projects one inch through the skin. This approximative estimate, it will be easily seen, could be made with almost mathematical accuracy were such precision necessary. The separation of the projecting fragment from the muscles with which it is naturally in contact, is measured by a pyramidal space whose summit is above and base below, represented by the thickness of the tibia at the seat of the fracture, and whose height is two or three inches. If reduction is not immediately effected, immediate union is impossible, and protracted suppuration occurs. After perfect reduction there will be internally a kind of cavity, bounded below by the bone and superficially by the skin; but the skin being perforated, there can be no accumulation of matter if the wound remains open, and if it becomes united and pus accumulates under the skin it will often escape by causing the wound to re-open, or if it does not, its presence is at once perceived and artificial exit is easily given to it. On the external side of the bone, on the contrary, if the separated parts do not immediately unite, the suppuration tends to increase the extent of their separation, and the pus, instead of escaping externally, even when the wound is open, spreads along the tibia, forming an extensive abscess, which is usually not perceived for some time.—*Ibid from Ibid.*

